# Chronic Urinary Infection in Overactive Bladder Syndrome: A Prospective, Blinded Case Control Study

**DOI:** 10.3389/fcimb.2021.752275

**Published:** 2021-09-30

**Authors:** Zainab Khan, Gareth D. Healey, Roberta Paravati, Nidhika Berry, Eugene Rees, Lavinia Margarit, Deyarina Gonzalez, Simon Emery, Robert Steven Conlan

**Affiliations:** ^1^ Swansea Bay University Health Board, Singleton Hospital, Swansea, United Kingdom; ^2^ Institute of Life Science, Swansea University Medical School, Swansea University, Swansea, United Kingdom; ^3^ Cwm Taf Morgannwg University Health Board, Obstetrics Gynecology Department, Princess of Wales Hospital, Bridgend, United Kingdom

**Keywords:** overactive bladder syndrome, subclinical infection, enhanced culture, bacteria, midstream urine culture

## Abstract

**Objectives:**

To investigate whether women with overactive bladder (OAB) symptoms and no evidence of clinical infection by conventional clean-catch midstream urine cultures have alternative indicators of sub-clinical infection.

**Patients/Subjects, Materials & Methods:**

The study was a prospective, blinded case-control study with 147 participants recruited, including 73 OAB patients and 74 controls. The OAB group comprised female patients of at least 18 years of age who presented with OAB symptoms for more than 3 months. Clean-catch midstream urine samples were examined for pyuria by microscopy; subjected to routine and enhanced microbiological cultures and examined for the presence of 10 different cytokines, chemokines, and prostaglandins by ELISA.

**Results:**

The mean age and BMI of participants in both groups were similar. No significant difference in the number of women with pyuria was observed between OAB and control groups (p = 0.651). Routine laboratory cultures were positive in three (4%) of women in the OAB group, whereas the enhanced cultures isolated bacteria in 17 (23.2%) of the OAB patients. In the control group, no positive cultures were observed using routine laboratory cultures, whereas enhanced culture isolated bacteria in 8 (10.8%) patients. No significant differences were observed in the concentrations of PGE2, PGF2α, MCP-1, sCD40L, MIP-1β, IL12p70/p40, IL12/IL-23p40, IL-5, EGF and GRO-α between the OAB and control groups.

**Conclusions:**

Patients with OAB symptoms have significant bacterial growth on enhanced culture of the urine, which is often not detectable through routine culture, suggesting a subclinical infection. Enhanced culture techniques should therefore be used routinely for the effective diagnosis and management of OAB.

## Introduction

Urinary incontinence (UI) is prevalent in society, affecting women of all ages and with potential to seriously impact the physical, psychological, and social wellbeing of affected individuals. According to the International Continence Society (ICS), overactive bladder (OAB) syndrome is characterized by urinary urgency usually accompanied by frequency and nocturia, with or without urgency urinary incontinence, and in the absence of urinary tract infection or other obvious pathology (Abrams et al., 2003; Haylen et al., 2010). As a major cause of urinary incontinence, OAB affects 12.8% of women over the age of 18 years of age and the incidence rises with age (Irwin et al., 2006). The incidence of any OAB symptom is as high as 48% (de Boer et al., 2011), however, there is a paucity of evidence associating OAB symptoms with urinary infection. OAB patients that are refractory to treatment are a particular challenge with the lack of a treatment response often attributed to an undiagnosed infection, despite this having been ruled out by urinalysis and standard culture, or poor drug efficacy.

The impact of OAB on quality of life can be significant. With adverse effects ranging from poor sleep quality to impaired emotional well-being to social disruption and difficulties in education and employment, several studies have highlighted the negative impact that this syndrome can have on patients’ lives (Stewart et al., 2003; Bartoli et al., 2010). Added to this are significant healthcare costs associated with disease management, which although difficult to accurately determine, are estimated to be around €5bn for major economies across Europe and up to $82bn in the United States (Reeves et al., 2006; Reynolds et al., 2016).

Despite the option for non-pharmacological interventions, such as lifestyle changes, behavioral therapy, and pelvic floor muscle training; most women with OAB will need drug treatment, which can include antimuscarinic drugs, anticholinergics, beta 3-receptor antagonists or botulinum toxins (Andersson et al., 1999; NICE, 2016a). In cases where drug therapy fails, additional management strategies including percutaneous posterial tibial nerve stimulation or sacral nerve stimulation, augmentation cytoplasty or urinary diversion can be considered. Although these are only indicated following extensive multidisciplinary team review and the failure of other treatment options (NICE, 2016b).

A key facet of the diagnosis of OAB is the absence of urinary tract infection (UTI), which is defined by urinary pathogens within a clean-catch midstream urine (MSU) sample and the presence of clinical symptoms. Although OAB and UTI share similar symptoms such as urgency frequency and nocturia, the onset of symptoms is different; UTI exhibiting acute symptoms and OAB exhibiting chronic symptoms. The similarity of sensory bladder symptoms associated with OAB and UTI can complicate disease management if UTI is not first excluded. Although there is no officially accepted standard for the minimum level of bacteria in a MSU specimen defining an infection, many laboratories use 10^5^ colony forming units (CFU)/mL urine as the UTI threshold (Kass, 1957), whilst others have proposed a threshold of ≥10^2^ CFU/ml in symptomatic women presenting with dysuria and cystitis-like symptoms (Stamm, 1983). Conventional MSU culture judged positive at ≥10^5^ CFU/ml can fail to detect 50% of all clinically significant, symptomatic urinary infections in OAB patients (Khasriya et al., 2008). As symptoms of OAB and cystitis overlap, a negative MSU is not entirely reliable.

The aim of the present study was to investigate whether urine from women with OAB symptoms (and no evidence of clinical infection by conventional MSU) have significant markers of sub-clinical infection when compared to individuals with no OAB symptoms.

## Patients/Subjects, Materials and Methods

The study was a prospective, blinded case control study approved by the Southwest Wales Research Ethics Committee (REC reference number 12/WA/0405), performed at Singleton Hospital, Swansea, and Swansea University Medical School from 2012 to 2015. The objectives of the study were to discover whether patients with OAB exhibit significant pyuria on fresh microscopy, harbor bacteria undetected by routine MSU cultures, and if so, to identify and quantify such bacteria. In parallel, we determined whether these patients displayed elevated levels of inflammatory markers.

147 subjects were recruited: 73 OAB patients and 74 controls. Inclusion criteria were female patients of at least 18 years of age presenting with OAB symptoms for more than 3 months. Symptoms were defined as greater than 1 episode/week of an uncontrolled urge to void causing incontinence. Exclusion criteria included hematuria, obstructive uropathy, vaginitis, urothelial carcinoma, urinary tract infection, pelvic radiation disease, neurogenic bladder, women with predominant stress urinary incontinence, liver disease, patients receiving antibiotics, an inability to give consent and understand English, and participation in other studies where the intervention is likely to affect their condition. The control group was recruited on a voluntary basis and comprised women aged at least 18 years of age, with no history of urinary infection, OAB symptoms or urge incontinence.

All participants completed a study specific Proforma and International Consultation on Incontinence Questionnaire-Overactive Bladder (ICIQ-OAB) (Avery et al., 2004). Information was obtained on patient’s age, BMI, past medical history, co-morbid conditions, urinary symptoms, prolapse symptoms and medication history. The ICIQ-OAB is a validated questionnaire (https://www.baus.org.uk/_userfiles/pages/files/Patients/Leaflets/ICIQ-OAB.pdf) used to screen for overactive bladder. It comprises 4 questions pertaining to frequency of micturition, nocturia, urgency and urge urinary incontinence. Each question is scored from 0 to 4 depending on the presence and severity of symptoms. The overall score of the ICIQ-OAB questionnaire ranges from 0-16 with greater values indicating increased symptom severity.

### Sample Collection and Processing

Participants provided an MSU specimen that was immediately divided into four equal aliquots. Aliquot one was analyzed directly for pyuria (white cell count ≥10^3^ CFU/ml) using microscopy within one hour of collection in a KOVA slide hemocytometer (Perera, 1985; Emerson and Emerson, 2005). Enhanced culture was carried out by inoculating 0.001 ml of urine onto chromogenic (trypticase soy agar with 5% sheep blood), chocolate, and fastidious anaerobic agar (FAA) culture plates within one hour of sample collection. The chromogenic and chocolate agar plates were incubated aerobically for 5 days at 35-37°C. The anaerobic plate was incubated anaerobically at 35°C for 5 days. Cultures were quantitated, and microorganisms isolated in the range of ≥10^3^ CFU/ml were identified. Bacterial colonies were identified by color change and size, and identification subsequently confirmed by matrix assisted laser desorption ionization-time of flight mass spectrometry (MALDI-TOF MS). Identification of microbes by MALDI-TOF MS was achieved either by comparing the peptide mass fingerprint (PMF) of the organisms with the PMFs contained in a database, or by matching the masses of biomarkers of organisms with a proteome database (Singhal et al., 2015). MSU samples containing one or two known uropathogens at ≥10^3^ CFU/ml were reported as enhanced culture positive. Specimens containing three or more isolates were reported as contaminated.

Aliquot 2 was transferred to a sterile MSU boric acid bottle and analyzed using an automated urine microscopy analyzer (IRIS 2000, Beckman Coulter, High Wycombe, UK) (Ben-Ezra et al., 1998; Ottiger & Huber, 2003). Particle counts were measured per unit of volume based on image number and volume scanned, and positive samples (single microbial species at a density of ≥10^5^ CFU/mL) were cultured, either fresh or after overnight storage at 4°C on chromogenic plates for 24 h at 35°C. The presence of multiple growths or micro-organisms <10^5^ CFU/mL are suggestive of urethral or vaginal contamination and are either reported as mixed growth or negative culture.

Aliquot 3 was snap-frozen and stored at -80°C prior to determining the levels of Prostaglandin E2 (PGE2, Abnova, Taiwan), Prostaglandin F2 alpha (PGF2 α, Abnova), Monocyte Chemotactic Protein-1 (MCP-1, R&D Systems, UK), Soluble fraction of the CD40 Ligand (sCD40L, R&D Systems), Macrophage inflammatory protein-1b (MIP-1b, R&D Systems), Interleukin 12p70/p40, IL-12p70/p40, R&D Systems), Interleukin 5 (IL-5, R&D Systems), Epidermal Growth Factor (EGF, R&D Systems), and Growth-Related Oncogene-alpha (GROα, RayBiotech, USA) using commercially available Enzyme-Linked Immunosorbent Assay (ELISA) kits.

Aliquot 4 was stored in Singleton hospital microbiology laboratory for a five-year period with ethical approval for storage and future sample testing.

### Sample Size Calculation and Statistical Analysis

Sample size was calculated using results from a comparative study (Tyagi et al., 2010), and from a pilot study conducted by this group. A sample of 36 per group was necessary to ensure 5% significance at a power of 80%. The precautionary step of allowing for a 10% shortfall in recruitment and 10% missing data resulted in a minimum total sample size of 45. Statistical analyses were performed using the IBM SPSS version 20.0 software (IBM Corp, NY, USA). Parametric statistical tests were used wherever possible, but due consideration was given to the distributional requirements of these tests, and suitable alternatives used where necessary. We used the Mann–Whitney test for comparison between groups for interval scale data. Chi-squared and McNemar’s tests were used for analysis of nominal data. A p-value ≤ 0.05 was considered to indicate statistical significance.

## Results

### Demographics

A total of 74 controls and 73 OAB patients were included in the study. The two groups were matched for age and BMI. The mean age and BMI of participants in the control group was 53 years (range 38-77) and 26 kg/m^2^ (range 20-50) compared to 57 years (range 33-74) and 27 kg/m^2^ (range 20-43), respectively. The demographic and clinical data of both groups is shown in **Table 1**. The OAB group had a higher incidence of previous gynecological surgery, including stress urinary incontinence (SUI) surgery. OAB patients also had a higher incidence of prolapse symptoms, SUI surgery, and voiding difficulties.

**Table 1:** Demographic and clinical data in the OAB and control groups.

		Control (n = 74)	OAB (n = 73)	p-value
**Demographics**	Age Mean	53.9375	57.3889	0.117
	BMI mean	26.7973	27.9701	0.980
	SUI	15 (20%)	60 (82%)	<0.001
	Prolapse	6 (8%)	32 (44%)	<0.001
	Voiding dysfunction	30 (41%)	1 (1%)	<0.001
	Dysuria	7 (9%)	0 (0%)	<0.001
**Previous surgery**	All	10 (13.5%)	25 (34%)	<0.001
	TAH	6 (8.1%)	17 (23.3%)	–
	VH+- PFR	2 (2.7%)	3 (4.1%)	–
	PFR	3 (4.1%)	4 (5.5%)	–
	Manchester Repair		1 (1.4%)	–
**Previous incontinence surgery**	All	1 (1%)	5 (7%)	0.014
	TVT=1	1 (1.4%)	2 (2.7%)	–
	TVT-O/TOT=2		2 (2.7%)	–
	Bladder sling		1(1.4%)	–

Both groups were comparable for age and BMI but had significant differences in the presence of SUI, prolapse symptoms, voiding dysfunction, dysuria, and previous surgery. SUI, Stress urinary Incontinence; TAH, Total Abdominal Hysterectomy; VH± PFR, Vaginal hysterectomy ± Pelvic floor repair; PFR, Pelvic floor repair; TVT, Transvaginal tape; TVT-O, Transvaginal tape-obturator; TOT, Trans-obturator tape.

### ICIQ-Questionnaire and Symptomatic Treatment

The ICIQ-Questionnaire score analysis demonstrated significantly higher scores in the OAB group compared to controls (**Table 2**, p < 0.001), with the control subjects being completely asymptomatic and receiving no treatment. Over 50% (40/73) of OAB patients were undergoing interventions, including 10 (14%) undertaking bladder retraining and pelvic floor muscle exercises, 23 (32%) receiving anti-muscarinic therapy, 4 (5%) receiving a Beta-3 agonist, and 3 (4%) awaiting botulinum toxin bladder injections having failed medical therapy.

**Table 2:** ICIQ OAB scores in Controls and OAB patients.

	Overall score	Control (n = 74)	OAB (n = 73)	p-value
**Median OAB symptom score 0/16**	0/16	1	8	<0.0001
**Frequency**	0/4	0	1	<0.0001
**Nocturia**	0/4	0	2	<0.0001
**Urgency**	0/4	0	2	<0.0001
**Urge Incontinence**	0/4	0	2	<0.0001

### Direct Microscopy Analysis for Pyuria

No significant differences between groups (16.4% OAB and 13.5% control, p = 0.65 and p = 0.44, respectively) were noted in the number of pyuria positive (white cell count ≥10^3^ CFU/ml) samples, as determined by hemocytometry or IRIS 2000 microscopy. This suggests that while useful for most women with high-count UTI, pyuria is a weak marker of low-grade infections and its value in lower urinary tract symptom (LUTS) patients, including OAB patients, is unclear.

### Routine and Enhanced Urine Cultures

Positive samples, identified by IRIS 2000 microscopy analysis, were cultured on chromogenic media plate at 37°C for 24 hours. The 3 control samples, initially reported as positive, were subsequently reported as negative since two had growth in the range of 10^3^-10^4^ CFU/mL and one had mixed growth. For the 9 cultured OAB samples, 2 had mixed growth, 4 had growth in range of 10^3^ CFU/mL and were reported as negative, and 3 were reported as positive with *E. coli* growth present at >10^5^ CFU/ml.

Enhanced culture of all samples (73 OAB and 74 controls) resulted in the isolation of a significantly higher number (p = 0.017) of uropathogens from 23 (31.5%) OAB samples and 11 (14.8%) control samples. After excluding mixed growth cultures, 17 (23.2%) OAB and 8 (10.8%) controls were found to have one or two microorganisms present (**Table 3**), including *E. coli*, *Enterococcus faecalis*, *Morganella morganii*, *Proteus mirabilis*, coliform, *Streptococcus*, and *Staphylococcus* species (**Table 4**). Enhanced culture identified additional microorganisms in 14 (82%) samples that tested negative using routine culturing methods, with bacteria being isolated from the majority of MSU specimens and highlighting the potential limitations of routine methods for OAB screening. Samples were only reported as positive when known uropathogens were present, however interestingly, vaginal commensals including *lactobacilli*, *corynebacteria*, *gardnerella*, and *alpha-hemolytic streptococci* species were more common in control samples than OAB patient samples.

**Table 3:** Routine and enhanced culture results in controls and OAB groups.

	Control (n = 74)	OAB (n = 73)	p-value
**Routine Culture positive**	0 (0%)	3 (4%)	0.079
**Enhanced culture positive**	11 (14.8%)	23 (31.5%)	0.017
**Enhanced culture positive (excluding mixed growths)**	8 (8.1%)	17 (23.2%)	0.045

**Table 4:** Types of microorganisms cultured in both groups.

Group	Microorganism cultured	Number
**OAB** (n = 17)	10^7^-10^8^ *Escherichia coli*	4
	10^7^-10^8^ *Enterococcus faecalis*	2
	10^6^ coliform	2
	10^6^-10^7^ *Enterococcus faecalis +* 10^6^-10^7^ *Streptococcus agalactiae*	1
	10^8^ *E. coli +*10^6^-10^7^ *Streptococcus*	2
	10^6^-10^7^ *Morganella morganii +*10^7^-10^8^ *Streptococci*	2
	10^6^ coliform *+* 10^7^ *Streptococcus pneumoniae*	2
	10^6^ coliform *+*10^6^-10^7^ *Staphylococcus*	2
**Control** (n = 8)	10^6^ *Enterococcus faecalis*	1
	10^8^ *Enterococcus faecalis +* 10^6^-10^7^ *E. coli*	1
	10^6^-10^7^ *Proteus mirabilis*	1
	10^7^-10^8^ *Escherichia coli +* 10^7^-10^8^ *Streptococcus anginosus*	1
	10^7^-10^8^ *coli +* 10^6^-10^7^ *Staphylococcus haemolyticus*	1
	10^7^-10^8^ *Enterococcus faecalis +* 10^7^-10^8^ *Staph*	1
	10^6^ coliform *+ 10^6^ Streptococcus anginosus*	2

### Analysis of Prostaglandins, Inflammatory Chemokines, and Cytokines

An association between inflammatory biomarkers and OAB has previously been suggested. To explore this, we investigated whether levels of these markers were altered in MSU samples from OAB and control patients. No significant differences between the OAB and control samples were noted for any of the markers evaluated (PGE2, PGF2α, MCP-1, sCD40L, MIP-1β, IL-12p70, IL-12p40, IL-5, EGF, and GROα, **Table 5**), the mean values of which were within the normal physiological range for a healthy person.

**Table 5:** Concentrations (pg/ml) of inflammatory prostaglandins, chemokines, and cytokines in OAB patients and controls.

	OAB	Control	p-value
	Mean ± SD (pg/ml)	Mean ± SD (pg/ml)	
**EGF**	10352.26 ± 9967.72	11424.62 ± 11895.60	0.860
**MCP**	95.77 ± 531.20	95.79 ± 558.46	0.519
**CD40**	972.03 ± 515.95	1140.86 ± 948.71	0.938
**PG E2**	102.27 ± 61.46	124.06 ± 93.83	0.336
**PG F2A with dilution**	9963.58 ± 5491.48	12300.75 ± 6176.91	0.081
**IL-5**	0.74 ± 0.93	0.84 ± 0.67	0.247
**IL12 p70**	1.65 ± 2.28	1.04 ± 1.32	0.213
**GRO-alpha**	2.01 ± 3.12	1.40 ± 3.25	0.089
**MIP-1 B**	1.71 ± 1.91	3.31 ± 3.66	0.078
**IL12P40**	11.36 ± 11.49	10.38 ± 12.13	0.408

## Discussion

The significant impact of OAB on quality of life makes early diagnosis and effective treatment essential. Symptomatic similarity with other conditions and the unreliable nature of routine microbiological culture, complicates the diagnostic process and means there is a need for more optimized approaches. By exploiting enhanced culture techniques, we have demonstrated a significantly higher rate of bacterial infection in OAB (23.28%) patients compared to control subjects (10.8%), than that estimated *via* the routine testing typically used to exclude infection in urinary samples. Data that are consistent with earlier reports (Khasriya et al., 2008; Khasriya et al., 2009; Hilt et al., 2014). An increased prevalence, in the control group, of vaginal commensal bacteria, which are regarded as an important component of the vaginal mucosa defence against genital infections, was also noted. By acidifying the vaginal microenvironment, these bacteria inhibit the colonization of invading pathogens through competitive exclusion. Our data are consistent with such a role for commensal bacteria in providing genital track mucosa homeostasis in healthy women, and with the clinical picture (Thomas-White et al., 2018). Using routine culture methods, only 4% of OAB samples in the present study were positive for uropathogens, demonstrating its limitations, and the incidence of pyuria was similar between OAB patients and control subjects. Despite a lack of consistency in the literature regarding the role of pyuria as diagnostic in LUTS patients, with different studies recording white cell counts ranging from 58%-74% in patients with OAB (Malone-Lee et al., 2007) to 22%-42% in LUTS patients (Kim et al., 2006), pyuria is generally considered a weak marker of low-grade infections, which is consistent with our data.

In contrast to some other studies, and despite the detection of uropathogens by enhanced culture techniques. We did not find any significant increase in the inflammatory markers tested in this study, suggesting that the sub-clinical levels of bacteria detected had not triggered an inflammatory response. The presence of cytokines in the urine has been studied in various bladder and kidney disorders as it provides a non-invasive tool for inflammation (Ninan et al., 1999; Abdel-Mageed et al., 2003). However, there is inconsistency in the studies analyzing the role of inflammatory markers in OAB. Tyagi et al. analyzed the urine of 17 idiopathic OAB patients and eight asymptomatic controls for 12 different chemokines, cytokines, and growth factors in a prospective study finding elevated levels of nine of these markers in the urine of OAB patients (Tyagi et al., 2010). The small sample size, cross-sectional study design, lack of comorbidities matching, and age disparity between the OAB and control group, is a potential limitation of Tyagi’s study, however. Ghoniem et al. analyzed an array of cytokines in the urine of 20 OAB patients, 20 controls, and 16 patients with UTI in a prospective, single blind study. Consistent with our observations, most of the cytokines were not elevated in the urine of OAB patients compared to controls or UTI patients. In fact, certain cytokines were significantly downregulated in OAB (Ghoniem et al., 2011). Currently the induction of a cytokine response in OAB remains to be established, and their utility as biomarkers for OAB diagnosis does not appear useful.

Heterogeneous results are also apparent for urine prostaglandins. Significant elevation in PGE2 and PGF2α in OAB patients compared to controls has been reported by Kim et al. and Kang Jun Cho, although prostaglandin concentrations were not corrected for urine creatinine concentration (Kim et al., 2006; Cho et al., 2013). Corroborating our study, however, Hsin-Tzu Liu did not demonstrate any significant difference in urinary PGE2/Cr levels among patients with interstitial cystitis/bladder pain syndrome (IC/BPS), OAB or controls (Liu et al., 2010). Thus, the role of urinary prostaglandins in the diagnosis of OAB also appears limited. Whilst the literature supports a role for inflammation in the pathophysiology of the overactive bladder, with the presence of many inflammatory markers described in OAB, studies performed with the purpose of identifying specific inflammatory markers have not demonstrated consistency.

Together, our data and the observations of others (Khasriya et al., 2008; Tyagi et al., 2010; Hilt et al., 2014), demonstrate that bacterial infection cannot be reliably excluded by routine culture and that inflammatory markers are an unreliable indicator of infection. Enhanced techniques or other methods, such as the identification of bacterial DNA in urine appear to be needed in all patients presenting with symptoms of OAB. Approximately 40% of OAB patients either do not achieve an acceptable level of a therapeutic benefit or remain completely refractory to treatment with conservative measures and antimuscarinics (Giarenis et al., 2013). Based on our observations that OAB patients have a higher incidence of bacterial infection, as demonstrated by enhanced culture techniques, it is plausible that these patients have unidentified urinary infections. The eradication of these subclinical infections, identified through the enhanced culture method used in the current study, by treatment with antibiotics might lead to simultaneous improvements in OAB symptoms and should form the basis of a future clinical trial.

## Data Availability Statement

The original contributions presented in the study are included in the article/supplementary material. Further inquiries can be directed to the corresponding author.

## Ethics Statement

The studies involving human participants were reviewed and approved by Southwest Wales Research Ethics Committee (REC reference number 12/WA/0405). The patients/participants provided their written informed consent to participate in this study.

## Author Contributions

ZK undertook the experimental work and prepared the draft manuscript. GH compiled the final manuscript and data tables. RP undertook part of the experimental work. NB, ER, and SE provided clinical input and study support. LM provided clinical input, conceived the study, and contributed to manuscript preparation. DG and RC conceived the study and contributed to manuscript preparation. All authors contributed to the article and approved the submitted version.

## Funding

Swansea Bay University Health Board Research & Development Department. Medical Research Council UK Confidence in Concept grant (MC_PC_19053).

## Conflict of Interest

The authors declare that the research was conducted in the absence of any commercial or financial relationships that could be construed as a potential conflict of interest.

## Publisher’s Note

All claims expressed in this article are solely those of the authors and do not necessarily represent those of their affiliated organizations, or those of the publisher, the editors and the reviewers. Any product that may be evaluated in this article, or claim that may be made by its manufacturer, is not guaranteed or endorsed by the publisher.
